# The Involvement of RAGE and Its Ligands during Progression of ALS in SOD1 G93A Transgenic Mice

**DOI:** 10.3390/ijms23042184

**Published:** 2022-02-16

**Authors:** Natalia Nowicka, Kamila Szymańska, Judyta Juranek, Kamila Zglejc-Waszak, Agnieszka Korytko, Michał Załęcki, Małgorzata Chmielewska-Krzesińska, Krzysztof Wąsowicz, Joanna Wojtkiewicz

**Affiliations:** 1 Department of Human Physiology and Pathophysiology, School of Medicine, University of Warmia and Mazury, 10-082 Olsztyn, Poland; kamila.szymanska@uwm.edu.pl (K.S.); kamilazglejc@gmail.com (K.Z.-W.); agnieszka.korytko@uwm.edu.pl (A.K.); joanna.wojtkiewicz@uwm.edu.pl (J.W.); 2 Department of Animal Anatomy, Faculty of Veterinary Medicine, University of Warmia and Mazury in Olsztyn, 10-719 Olsztyn, Poland; michal.zalecki@uwm.edu.pl; 3 Department of Pathophysiology, Forensic Veterinary Medicine and Administration, Faculty of Veterinary Medicine, University of Warmia and Mazury in Olsztyn, 10-719 Olsztyn, Poland; malgorzata.chmielewska@uwm.edu.pl (M.C.-K.); wasowicz@uwm.edu.pl (K.W.)

**Keywords:** RAGE, ligands, ALS, SOD1 G93A, transgenic mice, neurodegeneration

## Abstract

Amyotrophic lateral sclerosis (ALS) is a fatal neurodegenerative disease characterized by a progressive degeneration of upper and lower motor neurons that causes paralysis and muscle atrophy. The pathogenesis of the disease is still not elucidated. Receptor for Advanced Glycation End Product (RAGE) is a major component of the innate immune system and has implications in ALS pathogenesis. Multiple studies suggest the role of RAGE and its ligands in ALS. RAGE and its ligands are overexpressed in human and murine ALS motor neurons, astrocytes, and microglia. Here, we demonstrated the expression of RAGE and its ligands during the progression of the disease in the transgenic SOD1 G93A mouse lumbar spinal cord. We observed the highest expression of HMGB1 and S100b proteins at ALS onset. Our results highlight the potential role of RAGE and its ligands in ALS pathogenesis and suggest that some of the RAGE ligands might be used as biomarkers in early ALS diagnosis and potentially be useful in targeted therapeutic interventions at the early stage of this devastating disease.

## 1. Introduction

Amyotrophic Lateral Sclerosis (ALS), also known as Motor Neuron Disease (MND), is an age-related progressive neurodegenerative disease characterized by a selective loss of neurons in the cortex, brainstem, and spinal cord. Most ALS patients die within 2–5 years after symptom onset, resulting from muscle paralysis and respiratory insufficiency [1,2]. The pathogenesis of the disease is still not fully elucidated. Until now, evidence indicated that a variety of factors such as enhanced oxidative stress, neuroinflammation, protein misfolding and aggregation, glutamate toxicity, mitochondrial dysfunction, and impaired axonal transport [3,4] might be involved. However, most recent data indicate that major factors contributing to ALS development might lie elsewhere. Most recent findings indicate that the loss of function and protein accumulation might be key in developing the disease. Studies showed that protein aggregates in neuronal cells had been linked to proteinopathy, present in prion and prion-like diseases affecting neuronal tissues. Protein aggregates have already been reported in a couple of neurodegenerative diseases such as Alzheimer’s, Parkinson’s, and Huntington’s, most recently in ALS [5].

The toxicity of protein aggregates might be a key mechanism in the pathogenesis of ALS. However, neurodegenerative changes may also be caused by a prion-like mechanism [6,7] similar to those observed in Alzheimer’s disease [8] and Creutzfeldt-Jacob’s disease [9], where RAGE, the receptor for advanced glycation end products, has also been demonstrated to play a role. Recent evidence showed a link between proteinopathy, oxidative stress, and mitochondrial dysfunction [10], all of these factors are also part of RAGE signaling pathways.

RAGE is a signal transduction receptor encoded by the AGER gene, first identified as a cell surface receptor for Advanced Glycation End-products (AGEs) [11,12]. In the last decade, it has been re-established as a multiligand, pattern recognition receptor activated by a multitude of molecules such as members of the S100/calgranulin family and High Mobility Group Box-1(HMGB1), oligomeric forms of Aβ, phosphatidylserine (PS), and lysophosphatidic [13] among others.

AGEs are products of non-enzymatic glycation and oxidation of proteins/lipids that accumulate in various pathological processes and disorders such as inflammation, diabetes, and neurodegenerative diseases like Alzheimer’s and ALS [14]. Protein glycation results from irreversible non-enzymatic modification of protein structure, and through that process, proteins gain the ability to signal through RAGE [15]. As a transmembrane/surface protein, RAGE does not have catalytic activity and needs to interact with other adaptor proteins known as its ligands to exert its action [16]. RAGE signaling determines cell destiny, triggering either neuronal death or survival depending on concentration, cellular context, and type of the ligand. Interestingly, RAGE is extensively expressed during fetal life, and its expression gradually decreases after birth. However, under pathological conditions linked to neuronal degeneration such as ALS, it is again expressed at a very high level [17].

Mechanism of RAGE-AGE contribution to neurodegeneration remains unclear. However, studies show that RAGE interactions with S100B and/or HMGB1 might be key in triggering neuroinflammation and oxidative stress by activating downstream regulatory pathways such as JAK-STAT or NF-κB, causing neuronal dysfunction and apoptosis [18,19]. 

HMGB1 regulates transcriptions of various sets of genes, including proinflammatory genes, and it is almost a ubiquitous component of chromatin. HMGB1 is a proinflammatory cytokine that may contribute to neurodegenerative diseases like ALS by activating TLR4 and RAGE on target cells. HMGB1 is actively released under inflammatory conditions by neurons and glial cells. HMGB1, after binding to RAGE and TLR4 by immune cells, contributes to the production of proinflammatory cytokines by (NF-κB) pathway [20,21]. 

S100b is a calcium-binding protein predominately expressed by glial cells, mainly by the Central Nervous System (CNS) astrocytes. It belongs to a multigenic family of mostly dimeric calcium-binding proteins, including over 20 members with different homology to each other concerning the amino acid level [22]. In ALS, it has been found to contribute to gliosis triggered by reactive astrocytes. Physiologically, the role of astrocytes is to provide homeostasis of CNS. Unfortunately, during pathological processes like neurodegenerative disorders, S100b expressed by astrocytes acts like DAMP (damage associated molecular pattern), releasing a high protein concentration [23] and triggering neuronal dysfunctions and apoptosis. 

Even though the pathogenesis of ALS is still unknown, it should be emphasized that many research programs are being carried out to find an effective treatment, with highly committed researchers striving to find a novel therapeutic approach aimed at halting the progression of this devastating disease [24,25,26]. The latest studies have revealed that 4-hydroxy 2-nonenol (HNE), a product of lipid peroxidation, may be involved in the progression of ALS [27,28,29].

In the present report, we studied the involvement of RAGE and its ligands in inflammation and neurodegeneration of motor neurons in familial ALS in the mouse model of the disease.

## 2. Results

### 2.1. Life Quality, Mobility and Lifespan of Mice with Congenital ALS

**Figure 1:**
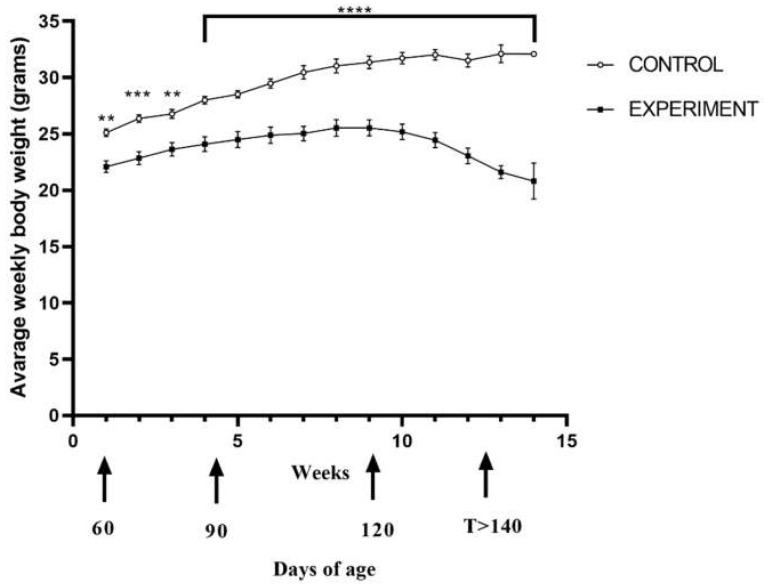
Body mass changes throughout the disease between ALS and control mice over time. All mice were weighed three times a week, beginning at eight weeks of age. The arithmetic weekly mean was taken from three weekly measurements: *n* = 7 in both groups. Statistical differences in body mass changes respectively ** 0.001 ≤ *p* ≤ 0.01; *** 0.0001 ≤ *p* ≤ 0.001; **** *p* ≤ 0.0001.

Mice from all groups were constantly monitored in terms of their physical condition and viability. In addition, mice from experimental groups were monitored for symptoms indicative of ALS, such as impaired mobility, paralysis of the limbs and difficulties with food intake. The onset of the disease, estimated by weight loss, occurred on average at about 100–110 days of age (Figure 1). It has been noticed that transgenic mice weighed less than the control group at the beginning of the experiment. That initial difference in body mass continued to increase throughout the experiment compared to the control group. For the first eight weeks of the experiment, both groups of mice continued to gain weight; however, following the onset of the disease, mice from the experimental group started to lose weight, and that process continued until the end of the study. The difference in mean weight between the control and experimental mice was statistically significant in the first, second, third and subsequent weeks (*p* = 0.0044; *p* = 0.0004; *p* = 0.0025 and *p* < 0.0001, respectively) (Figure 1). 

**Figure 2:**
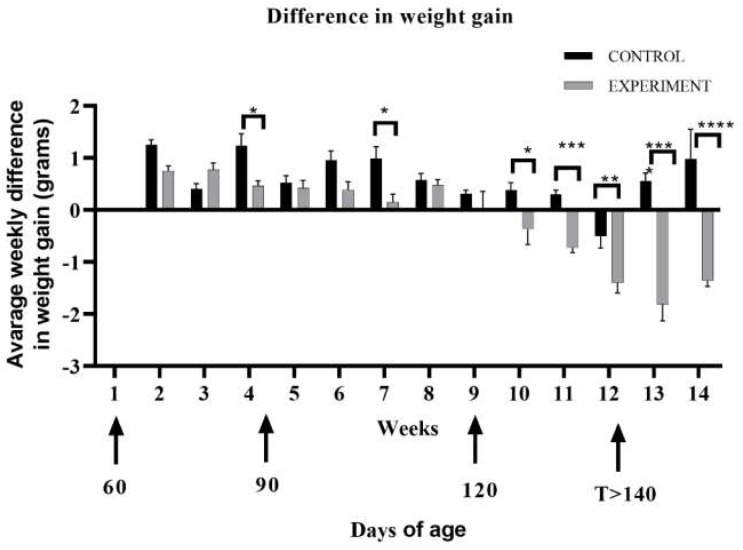
Increasing difference in the average weight gain between the control and experimental terminal group following the progression of ALS. Statistical differences in increasing difference in the average weight gain respectively * 0.01 ≤ *p* ≤ 0.05; ** 0.001 ≤ *p* ≤ 0.01; *** 0.0001 ≤ *p* ≤ 0.001; **** *p* ≤ 0.0001.

We observed that until 6 weeks into the study (until around 100 days of age), the rate of weight gain was similar in both the control and experimental group. However, after the sixth week of the experiment at the 120-day time point, mice from the experimental group began to lose their weight successively compared to the control group, which gained weight throughout the experiment (*p* = 0.0127; *p* = 0.0006; *p* < 0.0001, respectively) (Supplementary Materials Figure S1A,B). The observation was confirmed by a statistical analysis of the difference in weight gain between the control and experimental group. From tenth week of the study, the experimental group had negative weight gain as compared to control group (respectively * 0.01 ≤ *p* ≤ 0.05; ** 0.001 ≤ *p* ≤ 0.01; *** 0.0001 ≤ *p* ≤ 0.001; **** *p* ≤ 0.0001) (Figure 2). 

**Figure 3:**
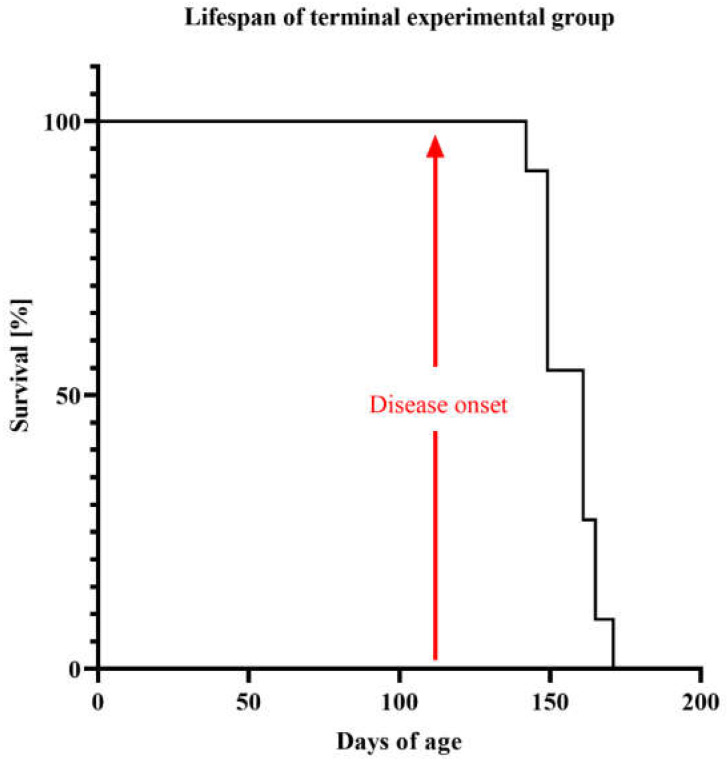
Kaplan–Meier curves showing survival probability of experimental terminal group. The primary endpoint was determined in accordance with critical points (weight loss greater than 20% of initial weight or the mouse lost its ability to right itself within 20 s). An arrow indicates disease onset.

We also showed the survival of the terminal experimental group, in which we could observe full ALS symptoms (Figure 3).

**Figure 4:**
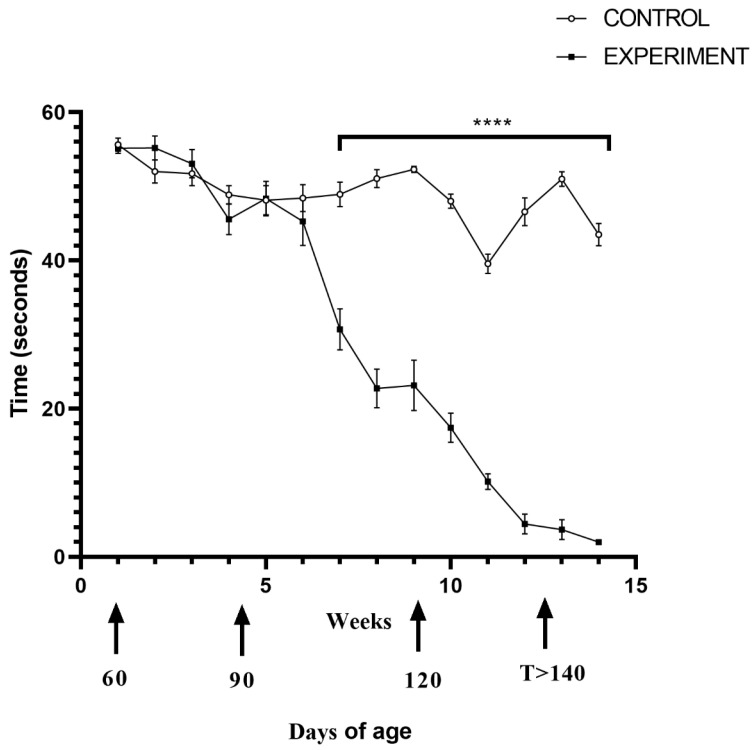
Results of hanging cage test in all groups 90, 120 and terminal experimental group as compared to the control. The test was performed twice a week. During each session, three sets of exercises were performed, with 30 s intermission between each test repetition. Statistical differences in hanging cage test respectively **** p ≤ 0.0001.

Motor performance score, measured by a hanging cage test coincided with the weight loss and revealed that the mouse ability to hold up during the test was weaker. Prior to the onset, ALS mice exhibited good motor performance scores; however, following the onset, the performance dramatically declined, and that decline continued until the end of the experiment *p* < 0.0001 (Figure 4). Around 110-days of age, mice from the experimental group started exhibiting significant difficulties in their ability to hold onto the wire mesh compared to the control group (*p* < 0.0001) (Figure 4).

### 2.2. mRNA Levels of RAGE and Its Ligands Transcripts in ALS Mouse Lumbar Spinal Cord

The analysis of amplified DNA sequences in examined tissue indicated over 96% homology with specific mouse sequences of *AGER*, *HMGB1*, *S100b,* and *IPO8* deposited in the GenBank database.

**Figure 5:**
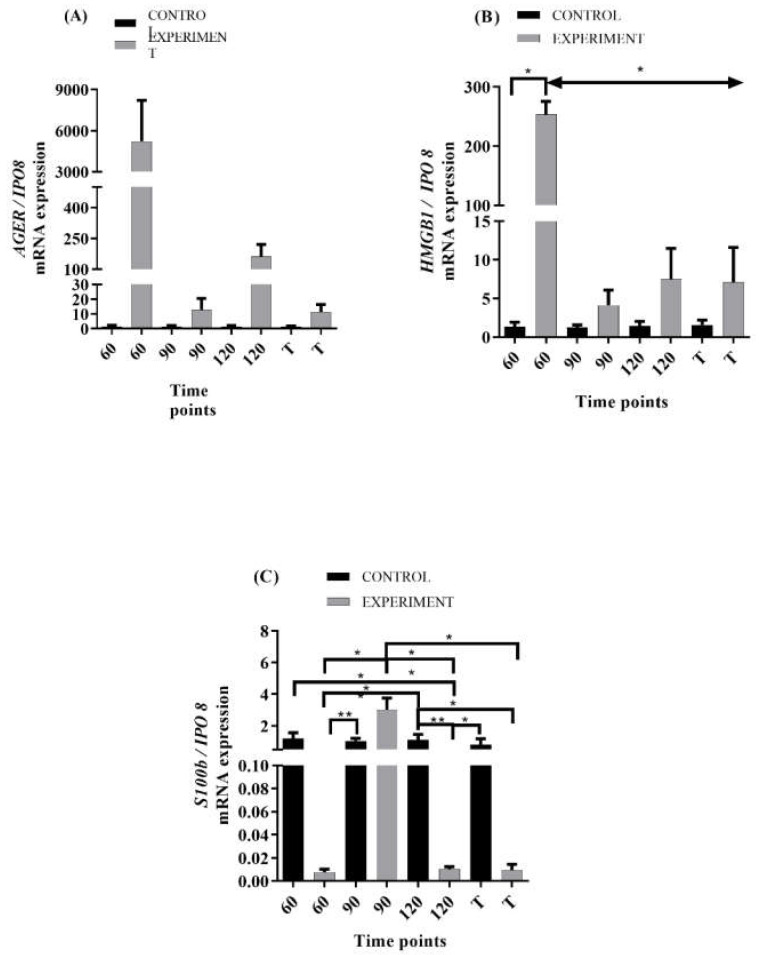
The relative expression of *AGER* (**A**), *HMGB1* (**B**), and *S100b* (**C**) mRNA in the lumbar spinal cord harvested from SOD1 transgenic mice with congenital ALS at baseline (60 days), 90, 120 days, and terminal time points. Data are presented as means ± S.E.M. relative to the geometric mean of the expression level of IPO8. Statistical differences in genes expression in the same tissues respectively * 0.01 ≤ *p* ≤ 0.05; ** 0.001 ≤ *p* ≤ 0.01. Abbreviation: ctr—control group; 90, 120, T—correspond to 90, 120 and terminal endpoints (experimental group); *n* = 6.

The relative expression of *AGER* (gene encoding RAGE) mRNAs in the experimental group at the 120-day time point differed significantly from the control group at the beginning of the experiment. (*p* ≤ 0.05, Figure 5A). We also observed that the expression was higher at the 120-day time point compared to a 90-day time point in the experimental group, terminal stage, and control group at the 90-time point, *p* = 0.0571. For the control group, the expression of *AGER* remained at a similar level throughout the whole study whereas in the experimental group, the highest expression of *AGER* was observed at 60-day time point *p* = 0.0902 and the lowest *p* = 0.1301 at the endpoint. The mRNA expression of *HMGB1* remained similar throughout the study in the control group, but differences were observed in the experimental group. In that group, the highest and significantly different expression was noticed at the beginning of the study compared to all other time points (0.01 ≤ *p* ≤ 0.05; Figure 5B). The expression pattern of *S100b* mRNA was different from *AGER* and *HMGB1,* with a high level of expression in the control group at all time points and lower levels of expression in the experimental group at all but 90-day time point. Statistical analysis revealed significant differences in *S100b* mRNA expression between control and experimental groups and within the experimental group alone (Figure 5C). Moreover, the reference gene expression did not differ statistically between the examined points and between the control and experiment groups.

### 2.3. Interaction Network of Studied Genes in ALS

**Figure 6:**
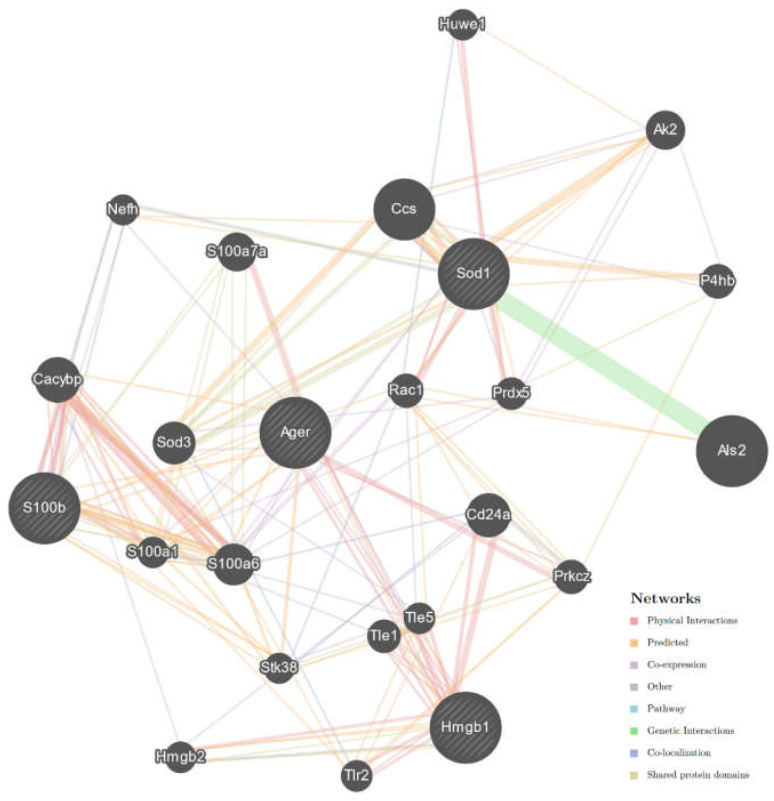
Interaction network constructed for *AGER* (the gene encoding RAGE), *S100B, HMGB1,* and SOD1 limited to *Mus musculus* (https://genemania.org/, accessed on: 3 August 2021). Studied (selected) genes are indicated with stripes. The interaction network was generated using GeneMANIA based on known and predicted interactions for studied genes. The colors of the lines suggest the type of interaction, i.e., red—physical interactions, purple—co-expression, orange—predicted, blue—co-localization, light blue—pathway, green—genetic interactions, olive—shared protein domains, gray—other. The GeneMANIA analysis indicated that *AGER* has the largest direct and indirect interactions count.

The GeneMANIA analysis interaction network of selected genes, i.e., *AGER*, *HMGB1*, *S100B,* and *SOD1* (*SOD1^G93A^* mice), indicated that they are all connected within one network (Figure 6). The network was created using 20 related genes with 24 total genes (including four studied genes) and 208 total links. Physical gene interactions were found in 54, co-expression in 32, shared protein domains in 25, co-localization in eight, other in two cases, and genetic interactions in one case (Supplementary Table S1). However, the GeneMANIA Prediction Server may predict interactions de novo without prior knowledge of existing interactions. Predicted interaction was found in 86 cases (Supplementary Table S1). *AGER* was the node with the largest count of direct and indirect interactions (11 interactions). The GeneMANIA analysis showed that *S100B* eight and *HMGB1* six-count of direct and indirect interactions.

### 2.4. Protein Levels of RAGE, Its Ligands, and 4HNE in ALS Mouse Lumbar Spinal Cord

**Figure 7:**
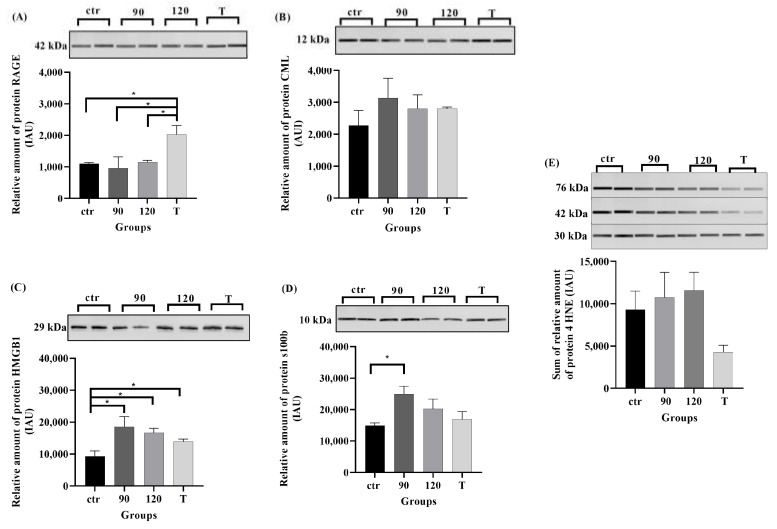
The relative amount of proteins: RAGE (**A**), CML (**B**), HMGB1 (**C**), s100b (**D**) and the sum of 4HNE (**E**) in the lumbar spinal cord harvested from SOD1 transgenic mice with congenital ALS at 90, 120, and terminal time points. Data are presented as means ± S.E.M. Statistical differences in relative amount of protein respectively * 0.01 ≤ *p* ≤ 0.05; Abbreviation: ctr—control group- 90 time point, *n* = 4; 90 *n* = 3, 120 *n* = 3, T *n* = 4—correspond to 90, 120 and terminal endpoints (experimental group).

The highest level of RAGE (42 kDa) was observed at the terminal stage of ALS compared to control, 90-day and 120-day time points (respectively *p* ≥ 0.05; 0.01 ≤ *p* ≤ 0.05; *p* ≥ 0.05; Figure 7A). The highest amount of HMGB1, CML, S100b was observed in ALS mouse lumbar spinal cord harvested at the 90-day time point (*p* ≤ 0.05 in all cases except for CML, Figure 7B–D) compared to the control. The relative amount of CML did not differ between the control and experimental group at any time point (*p* ≥ 0.05; Figure 7B). The relative amount of HMGB1 in the control group differed significantly as compared to the 90-day time point in the experimental group (0.01 ≤ *p* ≤ 0.05; Figure 7C), while the level of S100b was significantly different only between the control and experimental group at the beginning of the study (0.01 ≤ *p* ≤ 0.05; Figure 7D). The relative amount of 4HNE did not differ between the control group and all experimental groups (*p* ≥ 0.05; Figure 7E).

## 3. Discussion

Our study shows temporal changes in RAGE and its ligand expression from the onset to the terminal stage of ALS in a mouse model of the disease. The results of our study reveal an existing link between disease progression and the upregulation of RAGE and its signaling partners.

The results have shown elevated levels of RAGE encoding mRNA expression in ALS-model animals (at each stage of the disease) as compared to controls, suggesting the existence of some intrinsic mechanisms leading to overexpression of RAGE even before the disease onset, thereby confirming the potential role of RAGE in the pathogenesis of ALS [30,31,32]. Liu and co-workers (2020) indicated that inhibition of RAGE expression in ALS mice has a beneficial effect on their well-being and health status. Collectively, our results may suggest potential deleterious roles for RAGE in ALS and indicate that further testing of RAGE signal transduction pathways in ALS progression is warranted in this disease.

Interestingly, our results have revealed that the levels of mRNA expression of RAGE ligands differ, with S100b peaking at the onset of the disease as well as HMGB1 that, was at the highest level prior to the onset and followed by high and significant levels of expression later on throughout all remaining time points, as compared to controls. Surprisingly, in all groups the levels of HMGB1 mRNA expression reflected the expression of AGER. Protein levels of RAGE and its ligands partially corresponded to their mRNA levels, with RAGE being at the highest at the terminal stage of the disease and HMGB1 and S100b peaking at the onset of the disease. CML, a product of posttranslational processes and 4HNE, a lipid peroxidation product, thereby only detectable at the protein level, was shown in both cases as a stable pattern from the onset to the terminal stage of ALS in the mouse model of the disease. The observed minor discrepancy between protein and mRNA levels might arise from regulation by NF-κB (a nuclear factor kappa light chain enhancer of activated B cells). Once activated, the NF-κB transcription factor is responsible for an altered nuclear gene transcription or intracellular signaling involving AGER [33].

Furthermore, the changes between mRNA expression and protein level of RAGE and HMGB1 may be attributed to alternative splicing, posttranslational modifications, and protein stability [34]. It was found that there are many posttranslational modifications for both *AGER* and *HMGB1* genes. These mechanisms may contribute to differences between their mRNA and protein expression (https://www.genecards.org/cgi-bin/carddisp.pl?gene=AGER&keywords=RAGE; https://www.genecards.org/cgi-bin/carddisp.pl?gene=HMGB1&keywords=RAGE).

Moreover, the GeneMANIA analysis indicated that all studied genes are connected within one network in ALS mice. It may suggest that all these genes and encoded products may play a crucial role in ALS progression and can be useful as therapeutic targets for the development of therapeutics against ALS progression [35]. Furthermore, we found that AGER (the gene encoding RAGE) is a node of the genes network. It may suggest that RAGE is the main factor that modulates the progression of the disease and contributes to ALS-like pathology in male *SOD1^G93A^* mice.

Based on the results of our study, we might speculate that at the onset of ALS, when the accumulation of pathological changes exceeds motor neuron cells’ ability to self-repair, inflammatory and oxidative stress substances are being released in excess from immune cells activating RAGE and triggering a cascade of metabolic changes. RAGE signaling pathways lead to inflammatory cytokines and ROS release, causing structural protein damage and mitochondrial dysfunction. These changes combined with incorrect SOD1 functions lead to protein toxicity causing neuronal damage and contributing to neurodegenerative [36]. However, the precise understanding of the association between disturbed redox regulation and motor neuron death in ALS is unclear. To develop more successful therapies, establishing diagnostic biomarkers for early phase ALS would be required.

RAGE is one of the main components of the innate immune system, where HMGB1 is an endogenous ligand [32]. Here, we demonstrated an increased expression of HMGB1 in all experimental groups compared to the control. In mRNA expression of HMGB1, we observed the highest level at the beginning of the experiment as opposed to Lee et al., study, where the highest level of HMGB1 was observed in the end-stage mice [37]. Interestingly, the mRNA expression of HMGB1 is very similar to the expression level of AGER. Here, we hypothesize that the observed high level of HMGB1 at the 90 timepoint might be caused by excessive release of HMGB1 by activated microglia and astrocytes at the onset of the disease. During the progression of the disease, we also observed a reduction of HMGB1 in SOD1 G93A mice, likely due to neuronal degeneration [38]. It is known that HMGB1 binds to RAGE and toll-like receptors (TLR4). In turn, both receptors may signal through an independent pathway leading to NF-κB and activation of inflammatory cytokines [36]. An increasing body of evidence suggests that RAGE-HMGB1 interaction is strengthened during ALS [37]. This phenomenon indicated that HMGB1 might play a role in motor neuron injury and chronic, local inflammation progression in the ALS mouse model. We may suspect that HMGB1 may play a pathogenic role in ALS progression and maybe in future researcher directions.

The role of RAGE in the progression of ALS is well documented but still not fully understood. It is worth noting that RAGE binds to structurally diverse molecules, including proteins belonging to the S100 family [39]. It was reported that S100b might play a role in neuroinflammation observed in ALS [39]. In physiological conditions, S100b is released mainly by astrocytes. However, during pathological processes, secretion of S100b at high concentrations is considered to act as DAMP [25]. We have demonstrated that the protein expression of S100b and its mRNA transcripts peaked at the onset of ALS, followed by a progressive decline until the end of the experiment. This observation might indicate that the increase of S100b in astrocytes occurs at the pre-symptomatic stage of ALS [40]. Therefore it might be suggested that expression of S100b might be used as a pre-symptomatic marker [23] of the disease. Moreover, S100b has also been detected in serum patients with ALS [22,23,39,40]. However, further studies are necessary to explain the role of S100b as a potential ALS biomarker.

Conversely, RAGE is implicated in AGE accumulation [11,12]. Simultaneously, CML, N-carboxymethyl-lysine, is a typically advanced glycation end-product used to mark the level of AGEs in various pathological conditions and diseases [41,42]. Contrary to HMGB1 and S100b expression, we noticed stable CML protein levels in *SOD1^G93A^* mice. We hypothesize that a high, stable level of CML in experimental groups correlates with high protein glycation leading to neuroinflammation in ALS. The hyperactive RAGE signaling in ALS may also depend on RAGE binding to certain proinflammatory and regulatory ligands, such as CML [41,42]. Further studies are necessary to explain the role of RAGE-CML cross-talk in the progression of ALS. Nevertheless, the elevated expression of RAGE and its ligands might present both a potential mechanism and/or serve as a biomarker in ALS. One of the new concepts explaining the plausible mechanisms of ALS pathogenesis asserts that oxidative stress and accumulation of ROS is one of the main factors leading to its development. CML can interact with free amino groups in proteins, causing intracellular damage, cell death, and impaired cell functions resulting in age-related chronic diseases [43,44]. Simultaneously, studies show that CML and 4HNE may be involved in RAGE-triggered oxidative stress and inflammatory pathways by modulating NF-κB expression. Recent proteomic studies have revealed that 4HNE can create adducts with SOD1 protein [27,28,29]. The evidence indicates that 4HNE plays an essential role in oxidative stress and the progression of ALS. Studies demonstrated that in the spinal cord, the level of HNE is elevated in patients with ALS [27,28]. However, our results indicated that transgenic mice with progressing ALS disease lost weight. Consequently, the lipid peroxidation may be lower at the terminal point of the experiment than the time of onset of the disease. Moreover, studies show that 4HNE has a dual role; the lower level of HNE seems to be beneficial to cells and promote cell survival as well as proliferation. Contrary, the high level of 4HNE is harmful to cells and may lead to an elevated level of oxidative stress [27,28,29]. Nevertheless, further proteomic studies are necessary to elucidate the potential role of CML and 4HNE as a therapy target in patients with ALS. Additionally, oxidative stress generates accumulation and endogenous formation of AGEs like CML.

Furthermore, environment, metabolism, and lifestyle factors may play a role in the pathogenesis of ALS [45]. Hence, we can suspect that epigenetic modifications, i.e., DNA methylation, histone modification, miRNA expression, may regulate the expression levels of genes involved in ALS. Aberrant DNA methylation of genes in ALS may constitute a new direction for discovering mechanisms of ALS pathogenesis [45]. Moreover, the recent genome-wide study of DNA methylation in ALS identifies differentially methylated loci and implicates metabolic, inflammatory, and cholesterol pathways [46]. In view of these notions and our results demonstrating an increase in RAGE and its ligands levels, these concepts seem to be of particular importance.

It must be noted that those underlying molecular mechanisms of ALS are not yet completely examined and elucidated. However, the essential role of the immune system and inflammation in ALS pathogenesis has gained increased attention from researchers [32,37,39]. Our results indicated that RAGE and its ligands might have a crucial impact on ALS progression. Furthermore, the elevated level of HMGB1 and S100b at the onset of disease may suggest a crucial role of these proteins in the progression of ALS and early biomarkers of ALS. Consequently, HMGB1 and S100b may potentially impact ALS therapy [36,40]. Further studies are necessary to explain the role of HMGB1 and S100b proteins in ALS and indicate a new frontier of research.

## 4. Materials and Methods

### 4.1. Animals

All procedures during the experiment were performed according to the Local Ethical Committee of Experiments on Animals guidelines in Olsztyn, Poland (decision number 64/2018). In the study, 28 transgenic B6.Cg-Tg(SOD1*G93A)1Gur/J male mice, also known as B6 SOD1-G93A (stock number 004435; Jackson Laboratories, Bar Harbor, ME, USA), were used. Mice carrying the transgene exhibit paralysis resulting in a reduced life expectancy. According to The Jackson Laboratory (https://www.jax.org/strain/004435), transgenic mice have an abbreviated life span: 50% survive at 157.1+/−9.3 days in contrast to a control, non-transgenic group. As a control group, 28 C57BL6/J/cmdb male mice known as B6 (Medical University of Bialystok, Bialystok, Poland) were used. This inbred strain is the most widely used in biomedical research. We started the experiment with four-week-old animals. After two weeks of obligatory quarantine, all mice underwent a 2-week acclimatization and training period preparing them for a motor function test, i.e., hanging cage test. The beginning of the experiment was set when mice reached 60 days of age. Experimental and control animals were randomly divided into four groups of seven individuals per defined time points: 60, 90, 120, and the terminal stage of the disease (about 150 days, primary endpoint, natural demise). All mice were carefully weighted starting from the eighth week of age. The weight was recorded three times per week. Mice from three time point groups, i.e., 60, 90, and 120, were sacrificed independently at each of these time points regardless of their health status. The primary endpoint was determined when observed weight loss was greater than 20% of initial weight, or the mouse lost its ability to right itself within 20 s. All animals were sheltered in a designated Animal Facility at the University of Warmia and Mazury, Olsztyn, Poland, and kept in standard laboratory conditions. Following sacrifice, mouse lumbar spinal cord tissue samples were frozen in liquid nitrogen and stored at −80 °C for further analysis. 

### 4.2. Motor Function Test

The muscle strength test is a standard, well-established, and commonly used procedure in studies of motor neuron disorders. The test was performed twice a week; three sets of exercises were performed during each session, with 30 s intermission between each repetition of the test. All mice were placed in the center of the wire mesh screen (45 cm × 45 cm, 10 mm squares of 1 mm diameter wire), surrounded by 5 cm wooden slats to avoid injuring the mouse and its climbing to the other side of the screen. The screen was gently turned upside down about 30 cm above soft bedding. The time was noted after assigned 60 s or when the animal fell off on its own. The arithmetic mean was taken from three replicates for each exercise session. It was observed that at the beginning of the experiment, all mice were in good condition and were able to hold on to the wire mesh screen for 60 s. However, as the disease progressed, mice from the experimental group were losing the ability to hold on to the wire screen for 60 s, and the period they were able to stay attached to the screen was reduced, measured as a fall latency before the 60 s cut off. 

### 4.3. RNA and Protein Extraction

Total RNA and protein from the same lumbar spinal cord neuromere in each animal studied were isolated using the AllPrep DNA/RNA/Protein Mini Kit (Qiagen, Hilden, Germany) according to the manufacturer’s protocol. The quantity and quality of RNA were verified spectrophotometrically using Thermo Scientific^TM^ NanoDrop ^TM^ 2000/2000c Spectrophotometer (Thermo Fisher Scientific, Waltham, MA, USA). The protein concentration was determined using Direct Detect^®^ Infrared Spectrometer for Total Protein Quantitation (Merck Millipore, Darmstadt, Germany). 

### 4.4. Reverse Transcription and Quantitative PCR

One microgram of RNA was transcribed into cDNA by The QuantiNova Reverse Transcription Kit (Qiagen, Hilden, Germany) according to the manufacturer’s instructions. The obtained cDNA was used for quantitative PCR analysis (Table 1). The expressions of mRNA transcripts of genes encoding receptor for advanced glycation end-products *(AGER)*, high mobility group box 1 (*HMGB1*), S100 calcium-binding protein B (*S100b*) were investigated in duplicate by SYBR^®^ Green PCR Master Mix (Qiagen, Hilden, Germany) and a LightCycler^®^ 480 Instrument II (Roche Molecular Systems, Inc., Basel, Switzerland). The initial denaturation at 95 °C for 10 min was followed by 40 cycles of denaturation at 95 °C for 5 s, primer annealing at 62 °C for 10 s, and elongation at 72 °C for 10 s. All amplifications were followed by dissociation curve analysis of the amplified products. The relative expression of mRNAs was calculated by the ΔΔCt method and normalized using the geometric mean of expression levels of the housekeeping gene, i.e., importin 8 (*IPO8*), the more accurate gene measuring gene expression in the lumbar spinal cord [27]. In addition, PCR products were separated by electrophoresis on 2% agarose gel to verify them. Visualization of the products and images of gels were performed by Gel Imaging System (Bio-Rad, Hercules, CA, USA). The putative RAGE, HMGB1, S100b, SOD1, and IPO8 amplicons were isolated from the gel (Zymoclean Gel DNA Recovery Kits, Zymo Research, Hercules, CA, USA) and sent for sequencing to Genomed (Warsaw, Poland). Subsequently, the obtained DNA sequences were compared with those deposited in the GenBank database. 

### 4.5. Molecular Interactions between Studied Genes in ALS

The GeneMANIA Prediction Server (http://genemania.org, accessed on 3 August2021) was used to create an interaction network of studied genes, i.e., *AGER*, *HMGB1*, *S100B,* and *SOD1* in ALS [28]. The GeneMANIA Prediction Server provides a comprehensive set of functional annotation tools for investigators to understand the biological meaning of studied genes. The interaction network analysis was limited to *Mus musculus.*

### 4.6. Western Blot Analysis

Proteins (40 µg) were separated on 15-well 4–15% Mini-PROTEAN^®^ TGX™ Precast Protein Gels (Bio-Rad, Hercules, CA, USA) and transferred onto nitrocellulose membrane using a semi-dry system (Trans-Blot Turbo Transfer System, Bio-Rad). Prior to antibody incubation, the blotting membrane was blocked in EveryBlot Blocking Buffer (Bio-Rad) for 5 min at room temperature (RT). Primary antibody solutions were diluted in SignalBoost™ Immunoreaction Enhancer solution for primary antibodies (Merck Millipore, Darmstadt, Germany) and left for overnight incubation at 4 °C. The list of all primary antibodies is presented in Table 2. After four washes in PBS with 0.1% Tween-20, the membrane was incubated for one hour at RT with the corresponding fluorescence-labeled secondary antibody diluted in SignalBoost™ Immunoreaction Enhancer solution for secondary antibodies (Merck Millipore, Darmstadt, Germany). The bands were visualized with ChemiDoc Imaging Systems (Bio-Rad Hercules, CA, USA). Images were quantified densitometrically with ImageJ Software 1.50i (Wayne Rasband, National Institutes of Health, Bethesda, MD, USA) and compared between and within each group—control versus experimental, after normalization to the total amount of protein loaded in the gel.

### 4.7. Statistical Analysis 

All data were expressed as means with ± SEM. The normality and lognormality test (Shapiro-Wilk test) was performed before the statistical analysis.

The motor function test and average weight loss were compared between the control and experimental groups using parametric two-way ANOVA with Bonferroni post-hoc test.

The mRNA expression of RAGE, HMGB1, and S100b was compared between different time points using a nonparametric U Mann- Whitney test. The S100b expression was compared between all groups using the unpaired t-student test.

The relative amounts of proteins (RAGE; HMGB1; CML; SOD1; 4-HNE) tested in Western Blot were compared by one-way ANOVA with Tuckey’s post- hoc test.

The statistical significance of differences (*p* ≤ 0.05) was evaluated by GraphPad Prism 8.4.2. La Jolla, CA, USA.

## 5. Conclusions

In summary, our study is the first to report the expression of RAGE and its ligands over the full course of the disease. The findings of our study suggest that RAGE and its ligands may play a significant role in ALS pathogenesis and indicate that it might be useful to focus future research on the early stage of the disease when the expression of RAGE ligands, such as Hmgb1 and S100b is at the highest level in ALS mice.

## Figures and Tables

**Table 1:** Primers used for real-time PCR analysis.

Gene Symbol (Official)	Primer Sequences	Target Sequence Accession Number	Amplicon Length
AGER	F: 5′-CTTAGCTGGCACTTAGATGG-3′ R:5′-GAAACTGCAGGAGAAGGTAG-3′	L33412.1	168 nt
HMGB1	F: 5′-GAGAAGGATATTGCTGCCTAC-3′ R: 5′-CTTCATCTTCGTCTTCCTCTTC-3′	U00431.1	160 nt
S100b	F: 5′-GTCAGAACTGAAGGAGCTTATC-3′ R: 5′-CATGTTCAAAGAACTCATGGC-3′	NM_009115.3	185 nt
IPO8	F: 5′-CTATGCTCTCGTTCAGTATGC-3′ R: 5′-GAGCCCACTTCTTACACTTC-3′	NM_001081113.1	173 nt

**Table 2:** Antibodies used for Western Blot analysis.

Primary Antibodies						
Antigen	Code	Species		Working Dilution	Supplier	
RAGE	ab37647	Rabbit		1:1000	Abcam, Cambridge, UK	
HMGB1	ab18256	Rabbit		1:1000		
S100b	ab52642	Rabbit		1:1000		
CML	ab27684	Rabbit		1:5000		
4HNE	Ab46545	Rabbit		1:5000		
**Secondary Antibody**						
**Reagents**	**Code**		**Working Dilution**			**Supplier**
StarBright Blue 700 Goat Anti-Rabbit IgG,	12004162		1:10,000			BioRad Hercules, CA, USA

## Data Availability

All data supporting reported results is stored locally at University of Warmia and Mazury servers.

## Supplementary Materials

The following supporting information can be downloaded at: https://www.mdpi.com/article/10.3390/ijms23042184/s1.
