# Supplementary material for: The Involvement of RAGE and Its Ligands during Progression of ALS in SOD1 G93A Transgenic Mice
Source: Int J Mol Sci. 2022 Feb 16;23(4):2184. doi: 10.3390/ijms23042184 (PMC8880540; doi:10.3390/ijms23042184)
Supplement: Supplementary file 1 [file ijms-23-02184-s001.zip › ijms-1573451-supplementary.pdf]

Supplementary **Table S1**.

Molecular interactions between studied gene **–** GeneMANIA prediction server (http://genemania.org)

Gene 1	Gene 2	Interaction
Nefh	*S100b*	Co-expression
Rac1	*S100a6*	Co-expression
Nefh	*S100b*	Co-expression
Prdx5	*Ak2*	Co-expression
Tle1	*S100b*	Co-expression
S100a6	*Sod1*	Co-expression
Prdx5	*Ak2*	Co-expression
S100a1	*Ager*	Co-expression
Stk38	*Tle1*	Co-expression
Rac1	*S100a6*	Co-expression
Hmgb2	*Cacybp*	Co-expression
Sod3	*Ager*	Co-expression
Ak2	*Ccs*	Co-expression
S100a6	*Ager*	Co-expression
Tle5	*Ager*	Co-expression
Prkcz	*Cd24a*	Co-expression
S100a1	*Prdx5*	Co-expression
Prdx5	*Sod1*	Co-expression
S100a1	*Tle5*	Co-expression
Nefh	*S100b*	Co-expression
Prdx5	*S100a6*	Co-expression
Tle5	*Ager*	Co-expression
Hmgb2	*Cd24a*	Co-expression
Ak2	*Ccs*	Co-expression
P4hb	*Ccs*	Co-expression
P4hb	*Ak2*	Co-expression
Nefh	*S100b*	Co-expression
Tle5	*Sod3*	Co-expression
Nefh	*Ager*	Co-expression
Tle5	*Rac1*	Co-expression
S100a6	*Sod1*	Co-expression
Prdx5	*Sod3*	Co-expression
S100a6	*Cd24a*	Co-localization
Huwe1	*Rac1*	Co-localization
Nefh	*S100b*	Co-localization
Hmgb2	*Hmgb1*	Co-localization
Stk38	*Cd24a*	Co-localization
Tlr2	*Sod3*	Co-localization
S100a1	*S100a6*	Co-localization
Stk38	*Rac1*	Co-localization
Als2	*Sod1*	Genetic Interactions

Prkcz	*Cd24a*	Other
Nefh	*Sod1*	Other
Cd24a	*Hmgb1*	Physical Interactions
Cd24a	*Hmgb1*	Physical Interactions
S100a6	*Cacybp*	Physical Interactions
S100a7a	*Ager*	Physical Interactions
Ager	*Hmgb1*	Physical Interactions
Cacybp	*S100b*	Physical Interactions
S100a6	*Cacybp*	Physical Interactions
Prkcz	*Ager*	Physical Interactions
Rac1	*Sod1*	Physical Interactions
Tle1	*Hmgb1*	Physical Interactions
Prdx5	*Sod1*	Physical Interactions
Tlr2	*Hmgb1*	Physical Interactions
Tle5	*Hmgb1*	Physical Interactions
Huwe1	*Sod1*	Physical Interactions
Ccs	*Sod1*	Physical Interactions
Cacybp	*S100b*	Physical Interactions
S100a6	*Cacybp*	Physical Interactions
Tle5	*Tle1*	Physical Interactions
S100a1	*Cacybp*	Physical Interactions
Ccs	*Sod1*	Physical Interactions
Cacybp	*S100b*	Physical Interactions
S100a6	*S100b*	Physical Interactions
S100a6	*Cacybp*	Physical Interactions
Rac1	*Sod1*	Physical Interactions
S100a1	*S100b*	Physical Interactions
Hmgb2	*Hmgb1*	Physical Interactions
Ccs	*Sod1*	Physical Interactions
Cacybp	*S100b*	Physical Interactions
S100a6	*Cacybp*	Physical Interactions
Rac1	*Sod1*	Physical Interactions
Tlr2	*Hmgb1*	Physical Interactions
Ager	*Hmgb1*	Physical Interactions
S100a6	*Cacybp*	Physical Interactions
Tle1	*Hmgb1*	Physical Interactions
Tle5	*Hmgb1*	Physical Interactions
Cacybp	*S100b*	Physical Interactions
S100a6	*Cacybp*	Physical Interactions
S100a1	*Cacybp*	Physical Interactions
Tle1	*Hmgb1*	Physical Interactions
Tle5	*Hmgb1*	Physical Interactions
Ager	*Hmgb1*	Physical Interactions
S100a6	*Cacybp*	Physical Interactions
Tle1	*Hmgb1*	Physical Interactions

Tle5	*Hmgb1*	Physical Interactions
Ager	*Hmgb1*	Physical Interactions
Cacybp	*S100b*	Physical Interactions
S100a6	*Cacybp*	Physical Interactions
Prkcz	*Ager*	Physical Interactions
Rac1	*Sod1*	Physical Interactions
Tle1	*Hmgb1*	Physical Interactions
Prdx5	*Sod1*	Physical Interactions
Tlr2	*Hmgb1*	Physical Interactions
Tle5	*Hmgb1*	Physical Interactions
Huwe1	*Sod1*	Physical Interactions
S100b	*Ager*	Predicted
Cacybp	*Ager*	Predicted
S100a6	*Ager*	Predicted
S100a6	*Cacybp*	Predicted
Ak2	*Sod1*	Predicted
Ak2	*Ccs*	Predicted
Prkcz	*P4hb*	Predicted
Rac1	*S100b*	Predicted
Rac1	*Prkcz*	Predicted
Tlr2	*Prkcz*	Predicted
Tle5	*Tle1*	Predicted
S100a1	*Ager*	Predicted
S100a1	*S100b*	Predicted
S100a1	*Cacybp*	Predicted
S100a1	*S100a6*	Predicted
Hmgb2	*Hmgb1*	Predicted
Stk38	*Ager*	Predicted
Stk38	*Cacybp*	Predicted
Stk38	*S100a6*	Predicted
Stk38	*S100a1*	Predicted
S100b	*Ager*	Predicted
Ccs	*Sod1*	Predicted
Sod3	*Sod1*	Predicted
Sod3	*Ccs*	Predicted
S100a6	*S100b*	Predicted
Ak2	*Sod1*	Predicted
Tle5	*Tle1*	Predicted
S100a1	*Ager*	Predicted
S100a1	*S100b*	Predicted
S100a1	*S100a6*	Predicted
Hmgb2	*Hmgb1*	Predicted
Stk38	*Prkcz*	Predicted
Ccs	*Sod1*	Predicted
Ak2	*Sod1*	Predicted

Nefh	*Ccs*	Predicted
P4hb	*Sod1*	Predicted
Rac1	*Prkcz*	Predicted
Ccs	*Sod1*	Predicted
S100a6	*S100b*	Predicted
Ccs	*Sod1*	Predicted
S100a6	*S100b*	Predicted
S100a6	*Cacybp*	Predicted
Prdx5	*Sod1*	Predicted
Tle5	*Tle1*	Predicted
Huwe1	*Ak2*	Predicted
Stk38	*S100b*	Predicted
Ccs	*Sod1*	Predicted
Cacybp	*S100b*	Predicted
S100a6	*S100b*	Predicted
S100a6	*Cacybp*	Predicted
Stk38	*S100b*	Predicted
Ager	*Hmgb1*	Predicted
S100b	*Ager*	Predicted
Ccs	*Sod1*	Predicted
Cacybp	*S100b*	Predicted
S100a6	*S100b*	Predicted
S100a6	*Cacybp*	Predicted
Ak2	*Sod1*	Predicted
Prkcz	*Cd24a*	Predicted
Rac1	*Cd24a*	Predicted
Rac1	*Prkcz*	Predicted
Tle1	*Hmgb1*	Predicted
Prdx5	*Sod1*	Predicted
Tlr2	*Cd24a*	Predicted
Tlr2	*Sod3*	Predicted
Tlr2	*Prkcz*	Predicted
Tle5	*Hmgb1*	Predicted
Tle5	*Rac1*	Predicted
Tle5	*Tlr2*	Predicted
S100a1	*Ager*	Predicted
S100a1	*S100b*	Predicted
S100a1	*Sod3*	Predicted
S100a1	*S100a6*	Predicted
S100a1	*Tlr2*	Predicted
Hmgb2	*Hmgb1*	Predicted
Stk38	*S100b*	Predicted
Nefh	*Sod1*	Predicted
Ccs	*Sod1*	Predicted
P4hb	*Sod1*	Predicted

S100a6	*S100b*	Predicted
Rac1	*Sod1*	Predicted
Rac1	*Als2*	Predicted
Tle5	*Tle1*	Predicted
Ccs	*Sod1*	Predicted
Ccs	*Sod1*	Predicted
Rac1	*Als2*	Predicted
Ccs	*Sod1*	Shared protein domains
Sod3	*Sod1*	Shared protein domains
Sod3	*Ccs*	Shared protein domains
S100a6	*S100b*	Shared protein domains
S100a7a	*S100b*	Shared protein domains
S100a7a	*S100a6*	Shared protein domains
Prdx5	*P4hb*	Shared protein domains
Tle5	*Tle1*	Shared protein domains
S100a1	*S100b*	Shared protein domains
S100a1	*S100a6*	Shared protein domains
S100a1	*S100a7a*	Shared protein domains
Hmgb2	*Hmgb1*	Shared protein domains
Stk38	*Prkcz*	Shared protein domains
Ccs	*Sod1*	Shared protein domains
Sod3	*Sod1*	Shared protein domains
Sod3	*Ccs*	Shared protein domains
S100a6	*S100b*	Shared protein domains
S100a7a	*S100b*	Shared protein domains
S100a7a	*S100a6*	Shared protein domains
Tle5	*Tle1*	Shared protein domains
S100a1	*S100b*	Shared protein domains
S100a1	*S100a6*	Shared protein domains
S100a1	*S100a7a*	Shared protein domains
Hmgb2	*Hmgb1*	Shared protein domains
Stk38	*Prkcz*	Shared protein domains

Suplementarny Figures

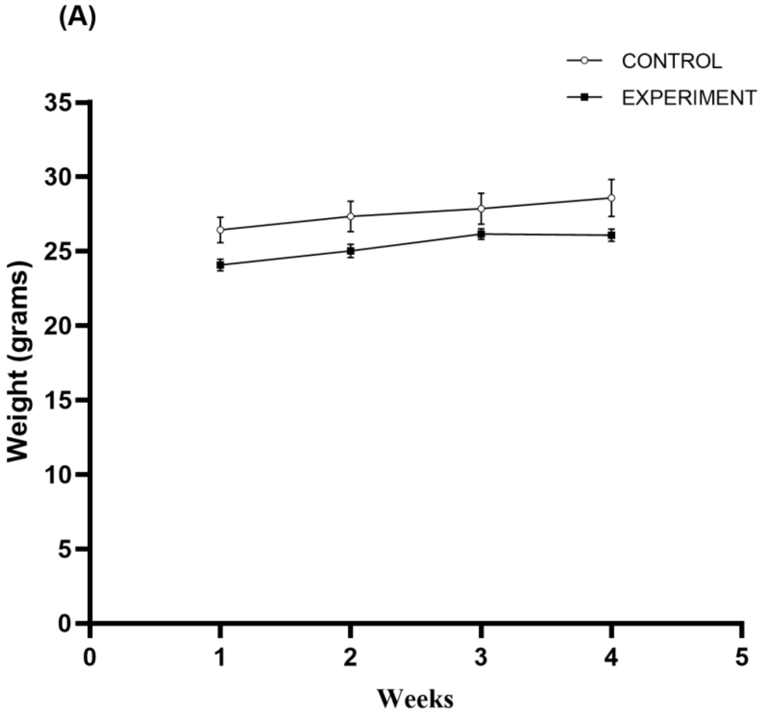

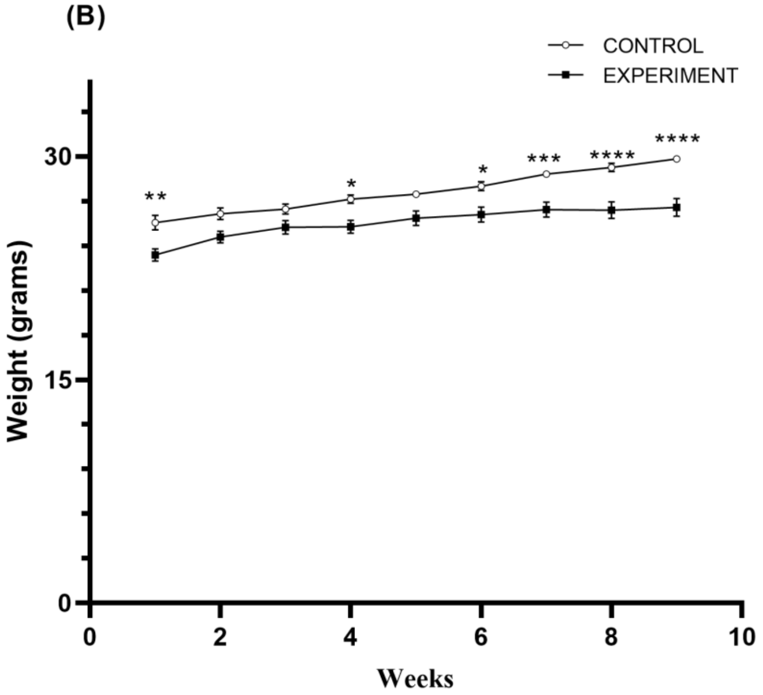

Figure S1 A-B Body mass changes over the course of the disease between ALS and control mice at 90 time point (A) and 120 time point (B). All mice were weighted three times a week beginning at eight weeks of age. The arithmetic weekly mean was taken from three weekly measurements: n=7 in both groups.
